# Statistically optimized pentazocine loaded microsphere for the sustained delivery application: Formulation and characterization

**DOI:** 10.1371/journal.pone.0250876

**Published:** 2021-04-30

**Authors:** Abdul Jabar, Asadullah Madni, Sajid Bashir, Nayab Tahir, Faisal Usman, Muhammad Abdur Rahim, Nasrullah Jan, Hassan Shah, Arshad Khan, Safiullah Khan

**Affiliations:** 1 Department of Pharmacy, The Islamia University of Bahawalpur, Bahawalpur, Pakistan; 2 College of Pharmacy, University of Sargodha, Sargodha, Pakistan; 3 Department of Pharmaceutics, BZ University, Multan, Pakistan; ISF College of Pharmacy, Moga, Punjab, India, INDIA

## Abstract

Pentazocine (PTZ) is a narcotic analgesic used to manage moderate to severe, acute and chronic pains. In this study, PTZ loaded Ethyl cellulose microsphere has been formulated for sustained release and improved bioavailability of PTZ. These microspheres were fabricated by oil in water emulsion solvent evaporation technique. A three factorial, three levels Box-Behnken design was applied to investigate the influence of different formulation components and process variables on the formulation response using the numeric approach through the design expert^®^ software. All the formulations were characterized for the morphology, different physicochemical properties and the results were supported with the ANOVA analysis, three dimensional contour graphs and regression equations. The maximum percentage yield was 98.67% with 98% entrapment of PTZ. The mean particle size of the formulations ranges from 50–148μm, which directly relates to the concentration of polymer and inversely proportional to the stirring speed. SEM revealed the spherical shape of PTZ microspheres with porous structures. These are physically, chemically and thermally stable as confirmed through Fourier transform infrared spectroscopy (FTIR), powder X-ray diffraction (PXRD) and thermal gravimetric (TG) analysis respectively. The microspheres provided a sustained release of the PTZ for more than 12 hours, following zero order with fickian and non fickian diffusion. The results indicate that prepared microspheres can be a potential drug delivery system (DDS) for the delivery of PTZ in the management of pains.

## 1. Introduction

Pain is an unpleasant sensory feeling associated with long term disability globally and regarded as the major health issue in society [1]. It is estimated that more than 21.5% of the total population was suffering from pain, including over 100 million patients in the USA and 14 million cases in the UK [2, 3]. Pain is an intricate physiological phenomenon, including various psychological and genetic origin described by various patients by their individual experiences. As pain is a perception and solely depends on the functioning of the cerebral cortex but not restricted to its any specific area [1]. Pain may be acute or chronic depending on the duration and reason for that pathophysiological condition. Acute pain often results from injury or disease and is the hostile, complicated, active physiological response to tissue trauma and various acute inflammatory processes. Pain is converted into chronic pain when it persists beyond the healing of the injury and the related inflammatory processes that usually persist for more than 3 months [4, 5]. Similarly, pain management in cancer therapy is an indispensable aspect. The cost of pain management is well developed, with a remarkable loss in productivity, increasing costs to the health care system and decreased quality of life [6]. Various strategies have been employed, but a wide consensus has been made on opioid base pharmacotherapy as first line strategy for treating moderate to severe pain with active diseases including cancer [7, 8]. Experimental models indicate that opioid analgesics regulate the perplexing variables to reduce experimental pain sensitivity across multiple stimulus modalities [9].

PTZ is an opioid analgesic and benzomorphane derivative that has been effectively used to manage acute and chronic pain associated with surgery and carcinogenesis [10, 11]. However, the oral administration of PTZ is associated with some drawbacks in terms of poor bioavailability (18–20%), short half-life (2–3 hours) and extensive first pass metabolism that required multiple dosing regimens to maintain the desired therapeutic levels. Interestingly, some of the physicochemical properties of the PTZ, such as Low molecular mass (285.4) and low oral bioavailability, suitable pKa values of 8.5 and 10 and log P of 2.0 make it an ideal candidate to formulate in a novel sustained release DDS such as microsphere which enhanced the therapeutic outcomes of the therapy by using PTZ [10, 12, 13].

In the last few decades, numerous methods have been used for the microencapsulation of drugs for sustained or controlled delivery; still, the effectiveness of these methods depends upon the quality of polymer or nature of the drug [14–16]. Previously, PTZ loaded proniosomes and niosomal gels have been prepared for topical administration that indicates improved permeability and dissolution rate [10]. Similarly, various DDS have been designed that have interrelated complex preparation steps and optimization processes. To identify the effect of these structural components and various process parameters, different mathematical models and statistical tools have been devised that payoff in terms of cost and time economy. Among these statistical tools, response surface methodology is considered prime important because it required a small number of lab trials that save time, chemicals and labor during the optimization process [17, 18].

Recently, various polymer based micro and nano-carriers have been developed to sustain the release of different therapeutic moieties at the site of absorption. Among these micro-carriers, microparticles, microcapsules and microspheres have been extensively studied. Microspheres are small spherical particles ranges from 1–1000 μm in diameter [19, 20]. There are different techniques for the formulation of microspheres including solvent evaporation technique [21], coacervation [22], phase separation, spray drying and spray congealing [23], and various polymerization techniques including normal polymerization, interfacial polymerization, quasi-emulsion solvent diffusion and polycondensation technique [24]. However, microspheres prepared by solvent evaporation emulsion have got the upper edge on other methods as they require moderate operating conditions [25, 26].

The present study aimed to design and optimized the PTZ loaded ethyl cellulose microspheres by oil in water emulsion solvent evaporation method using a design expert statistical tool. Ethyl cellulose was used as a polymer because of its natural origin and biocompatible nature with higher encapsulation potential for hydrophobic drugs. The effect of independent variables was accessed on the various responses such as particle size, entrapment efficiency (EE) and dissolution rate to obtain the optimized PTZ loaded microspheres. Furthermore, the optimized microspheres were characterized by different physicochemical properties and PTZ release.

## 2. Materials and methods

### 2.1. Materials

Ethyl cellulose was purchased from Sigma Aldrich, Germany. Pentazocine (Drug) was gifted by Global pharmaceuticals Pvt Ltd Islamabad Pakistan. Poly vinyl alcohol (PVA) was purchased from Sigma Aldrich Germany. All the solvents including dichloromethane (DCM), hydrochloric acid (HCl), sodium hydroxide and other solvents used were of analytical grade. Freshly prepared double distilled water was used throughout the experiment. All other chemicals used were of analytical grade.

### 2.2. Methods

#### 2.2.1. Experiment design for optimization of microsphere

The Design-Expert^®^ 7.0.0 employing the response surface methodology was designed to evaluate the effect of various independent variables on different formulation parameters. Three factors, three levels Box-Behnken design suggested 15 experimental runs were utilized for the optimization of the microspheres. The concentration of polymer (X1), Stirring speed (X2) and concentration of surfactant (X3) were considered as independent variables. Whereas, the particle size (Y1), entrapment efficiency (Y2), and dissolution rate (Y3) were considered as dependent variables. All the independent variables were selected at three different levels including the lower, middle and higher levels (-1, 0 and +1) as shown in Table 1. The optimization process was targeted to minimize the particle size, maximized entrapment efficiency and sustains the release of the microspheres.

**Table 1:** Levels of independent and dependent variables in design of experiment.

Independent variables		Levels			
		Maximum (+1)		Middle (0)	Minimum (-1)
**X1**	Polymer Concentration (mg)	150	100		50
**X2**	Stirring Speed (rpm)	450	300		150
**X3**	Surfactant Concentration (%)	1.0	0.75		0.50
**Dependent variables**		**Desired Outcomes**			
**Y1**	Particle Size (nm)	Minimized			
**Y2**	Entrapment Efficiency (%)	Maximized			
**Y3**	Dissolution rate (%)	In range			

The randomized selection was used for the preparation of various formulations from the given matrix of the 15 formulations to minimize the possibility of biases. The non-linear quadratic model expression (Eq 1) has been generated in this study design is given below

$$R_{1}=\mathrm{bo}+R1\;X_{1}+R2\;X_{2}+R3X_{3}+R12\;X_{1}X_{2}+R13\;X_{1}X_{3}+R23\;X_{2}X_{3}+R11\;X_{1}^{2}+R22\;X_{2}^{2}+R33\;X_{3}^{2}$$ (1)

Where, R is the response that we measured at each level of the independent variables in the experimental design; X1, X2 and X3 are the influencing variables at each level also designated as independent variables; b_0_ is the intercept; and R_1_–R_33_ were the regression coefficients of respective variables and their interactional terms calculated by experimental data. The factors X_1_X_2_, X_1_X_3_ and X_2_X_3_ indicate the interaction among the various parameters and $X_{1}^{2},\;X_{2}^{2}$ and $X_{3}^{2}$ represent the quadratic term of the equation [17].

#### 2.2.2. Preparation of PTZ loaded microsphere

The PTZ loaded Ethyl cellulose microspheres were prepared by oil in water emulsion solvent evaporation method reported by Kashif *et al*. [18]. The drug concentration was kept constant in all formulations (50 mg) while the polymer was taken in variant concentrations of 50, 100 and 150 mg, respectively, as suggested by design expert software. The internal phase was prepared by dissolving the drug and polymer in 5 mL DCM under gentle stirring. The aqueous phase was prepared by dissolving the varied concentrations of emulsifier (PVA) (0.5, 0.75, 1% w/v) in 50 mL distilled water and heated at 50°C on a hot plate magnetic stirrer. The internal phase was added in a dropwise manner in the aqueous phase and allowed to stir for 5 h. The organic solvent was allowed to evaporate from the mixture. Microspheres were filtered and washed several times (3 to 6) with 0.1 N HCl and finally with the water to remove the free drug. The microspheres were then dried in the freeze dryer (Christ alpha 1–4 LD, UK) and used for further investigation [27].

#### 2.2.3. Optimization of the formulation

Different statistical tools have been employed for the optimization of DDS in various studies. We employed the computer assisted process using Design-Expert^®^ software for the said purpose. In the optimization process, the provision of data about selected dependent variables of the formulation enables to predict the amount of those variables, such as concentration of polymer and surfactant as well as other process parameters for the preparation of optimized product The process also predicted the results of different dependent factors such as particle size, EE and the dissolution rate of the microspheres. The statistically suggested optimized formulation was prepared using the given variables and analyzed for morphology, different physiochemical properties and drug release kinetics modeling.

#### 2.2.4. Characterization of the microspheres

*2*.*2*.*4*.*1*. *Particle size of microspheres*. The particle size was measured by an imaging optical magnifying instrument (Eclipse E200-LED, Nikon, Tokyo, Japan) [28, 29]. The eyepiece micrometer was adjusted against the stage micrometer. The slide was prepared by sprinkling a small quantity of sample on the slide and fixing on x10 lens. Mean particle size was calculated by taking mathematical Eq 2.

$$\mathrm{Mean}\;\mathrm{Particle}\;\mathrm{size}=\frac{\mathrm{Sum}\;\mathrm{of}\;\mathrm{diameters}\;\mathrm{of}\;\mathrm{observed}\;\mathrm{microspheres}}{\mathrm{No}.\mathrm{of}\;\mathrm{observed}\;\mathrm{microspheres}}$$ (2)

*2*.*2*.*4*.*2*. *Morphological analysis of microparticles*. The morphology of completely dried PTZ loaded microspheres was performed using scanning electron microscope (Mel JEOL JSM-5910). A minute quantity of microspheres (powder form) of selected microsphere formulations (P3, P5 and P10) was poured on the double-sided carbon sticky tape. The extra amount of powder was shed off with the compressed nitrogen gas and the photomicrographs at different magnification different magnifications of 5kv ×120, 5kv ×150, 5kv ×250, 5kv ×270, 5kv ×350, 5kv ×400, 5kv ×600, 5kv ×800, 5kv ×900, 5kv ×2500, 5kv ×3000, 5kv × 5000 were taken [30].

*2*.*2*.*4*.*3*. *Percent yield*. The percent yield of all formulations was calculated to find the amount of recovery and wastage of the product during processing. The percent yield depends on the stickiness of the microspheres in powder form.

*2*.*2*.*4*.*4*. *Entrapment efficiency*. The direct method was utilized to find the EE. Accurately weighed 10 mg microspheres were dispersed in 100 mL of 0.1 N HCl. The dispersion was subjected to stirring for 12 h to achieve the ultimate disruption of the microsphere for complete extraction of the drug in the medium. The resultant dispersion was then filtered and the clear drug solution was obtained. 1 mL of the solution was taken and again diluted to 20 mL with 0.1 N HCl. The sample was then subjected to UV spectrophotometric analysis (IRMACO GmbH, Geesthacht Germany) at 278 nm to determine the amount of encapsulated drug [31].

Entrapment efficiency was calculated by Eq 3.

$$\mathrm{EE}\;\%=\frac{\mathrm{Drug}\;\mathrm{entrapped}}{\mathrm{Theoretical}\;\mathrm{drug}\;\mathrm{content}}X\;100$$ (3)

*2*.*2*.*4*.*5*. *Drug release and kinetics*. The *in vitro* drug release profile was performed by utilizing USP Type II dissolution mechanical assembly (Pharma test, Heinberg, Germany). The specific amount of microspheres containing 5 mg of the drug were suspended in 900 mL simulated gastric fluid (pH 1.2) as a dissolution media. During the 12 h release study, 5 mL sample was withdrawn periodically at predetermined time intervals and replenished with 5 mL fresh dissolution medium to keep up the sink volumes. Samples were measured using a UV spectrophotometer (IRMACO GmbH, Geesthacht, Germany) at 278 nm. Percent cumulative drug release against time was justified graphically with an optimum time interval (12 h) by Box-Behnken design [32]. The regression mathematical statement was used to compute the percent medication release.

$$Percent\;Drug\;Release=\frac{Qt}{Ql}*100$$ (4)

Kinetic analysis of *in vitro* release was accomplished to propound the order and mechanism of drug release. Coefficients of zero order release, first order release, Higuchi, and Korsmeyer-Peppas models were calculated using different formulas (Eqs 5–8) and estimated by regression analysis. Korsmeyer-Peppas model was deployed to resolve the drug release mechanism to ascertain the estimation of diffusion exponent ‘‘n” by fitting the drug release data.

Zero order kinetic model

$$A_{t}=-K_{0}t+A_{0}$$ (5)

First order kinetic model

$$\log A_{t}=-Kt/2.3+\log A_{0}$$ (6)

Higuchi model

$$F_{t}=K_{H}\times \sqrt{t}$$ (7)

Krosmeyer-Peppas model

$$\frac{\mathrm{Mt}}{M\infty }=Kt^{n}$$ (8)

*2*.*2*.*4*.*6*. *FTIR*. The interaction of the drug with the excipients was determined by FTIR spectroscopic analysis [33]. Pure PTZ, Ethyl cellulose, their physical mixture and formulation were analyzed in the range of 4000 to 400 cm^-1^ (Bruker tensor 27, Germany). Briefly, the powder sample was positioned at the ATR crystal and pressed in the face of the crystal by rotating and turning the arm to get efficient contact [34].

*2*.*2*.*4*.*7*. *Thermal analysis*. The thermal gravimetric analysis (TGA) was performed to evaluate the thermal stability of the formulations over an extended temperature range. Pure PTZ, Ethyl cellulose, their physical mixture and formulations were analyzed by (SDT Q600, TA Instrument Co., Ltd., America). Samples were fixed in aluminum containers and TGA runs were performed at a consistent rate of 10°C/min over a temperature scope of 0–500°C [35].

*2*.*2*.*4*.*8*. *PXRD analysis*. The crystallinity of the drug in pure PTZ, Ethyl cellulose, their physical mixture, and formulations was checked to explore the impact of microencapsulation. PXRD was performed at the D8 Advance BRUKER diffractometer. All the samples were placed on aluminum plates at ambient conditions and scans were performed at 2*θ* diffraction angle from 0 to 40° with a step size of 0.026° using nickel filtered Cu-Kα radiation. The applied voltage and current were set as 45 kV and 40 m [36].

### 2.3. Statistical analysis

The observed data were evaluated using dependent and independent variables. The response of the dependent variables was evaluated. The ANOVA was applied to determine and evaluate the significance, impact and effect of the independent variables. The effect was considered significant at *P*<0.05.

## 3. Result and discussion

### 3.1. Box Behnken design analysis and optimization

#### 3.1.1. Effect on particle size

The mean particle size of all the selected 15 formulations was in the range 50–148 μm depending upon the variation in the independent variables. Formulation P12 shows maximum particle size (148.16μm) while the minimum particle size was achieved in formulation P9 (50.53μm) as shown in Table 2. ANOVA analysis indicates that there was a significant effect of various independent variables on particle size. The quadratic equation of the selected model indicating the effect on above mentioned variables is given below.

$$\mathrm{Mean}\;\mathrm{particle}\;\mathrm{size}\;(Y_{1})=93.55+27.91\;X_{1}-18.60\;X_{2}-5.42X_{3}-3.89\;X_{1}X_{2}-1.09\;X_{1}X_{3}+3.27\;X_{2}X_{3}+4.62\;X_{1}^{2}-2.71\;X_{2}^{2}+3.39\;X_{3}^{2}$$ (9)

**Table 2:** Effect of formulation components and process variables on different characteristics of microspheres.

Formulation Code	*Particle size (μm)	%EE±S.D	% yield	Release % after 12 hrs.
P1	122.21±7.07	63.11±1.15	91.00±1.52	70.58
P2	102.57±7.58	90.26±0.53	75.33±2.08	75.21
P3	84.37±27.86	37.29±0.07	66.00±2.08	87.24
P4	79.33±4.47	57.64±0.18	71.33±2.51	89.22
P5	98.76±7.07	88.07±0.49	82.00±2.64	78.03
P6	92.66±20.41	54.16±0.18	93.33±2.05	81.84
P7	95.66±7.52	32.70±0.11	98.67±1.00	79.56
P8	68.75±4.47	30.46±0.14	76.00±0.57	96.91
P9	50.53±12.00	54.69±0.14	70.00±1.15	98.28
P10	136.54±9.17	67.50±0.14	76.50±1.52	78.92
P11	118.66±12.11	98.37±0.11	76.00±2.64	74.84
P12	148.16±14.97	89.05±0.24	70.00±0.57	63.12
P13	92.33±8.16	28.49±0.11	72.00±2.64	81.26
P14	76.33±16.25	70.38±0.11	68.67±2.05	95.82
P15	78.72±10.32	33.22±0.07	68.00±1.15	90.98

*Results are indicated as mean ± SD (n = 3).

**Fig 1:**
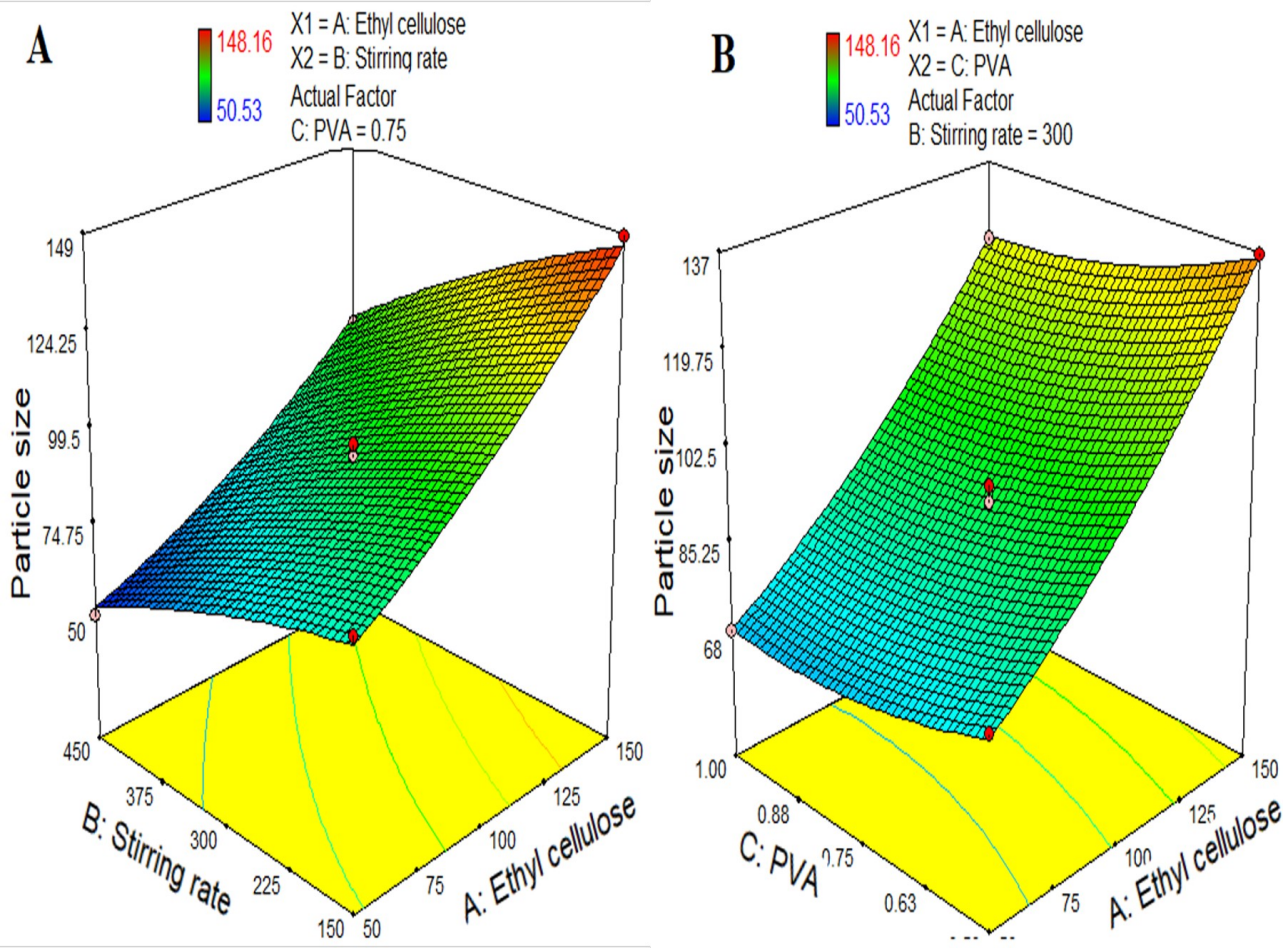
3 dimensional response surface graphs indicating (A) the effect of the concentration of polymer and stirring speed on particle size, and (B) the effect of the surfactant (PVA) concentration and stirring speed on particle size.

The statistical analysis indicates that there is a significant increase in the particle size (Y_1_) of the microsphere with an increase in the polymer concentration (X1) while the increase in the stirring speed (X2) and concentration of the surfactant (X3) decrease the particle size as indicated by the positive and negative coefficients in the regression equation (Eq 9) respectively. It was evident from Fig 1A that the particle size of the microsphere increases from 50 μm to 148 μm with an increase in the concentration of the ethyl cellulose from 50 mg to 150 mg that might increase the viscosity of the polymeric dispersion. Highly viscous dispersions are difficult to break into smaller particles as supported by the previous studies [37, 38]. However, higher stirring speed provides the shearing force for converting larger particles of the dispersion into smaller particles [39]. The higher stirring speed also decreases the viscosity of the dispersion contributing to smaller sized particles [40]. In addition, the concentration of surfactant might contribute to the change in the particle size. An increase in the surfactant results in the formation of the relatively compact matrix which will help to decrease the particle size of the prepared microsphere (Fig 1B) [41].

#### 3.1.2. Effect on entrapment efficiency

**Fig 2:**
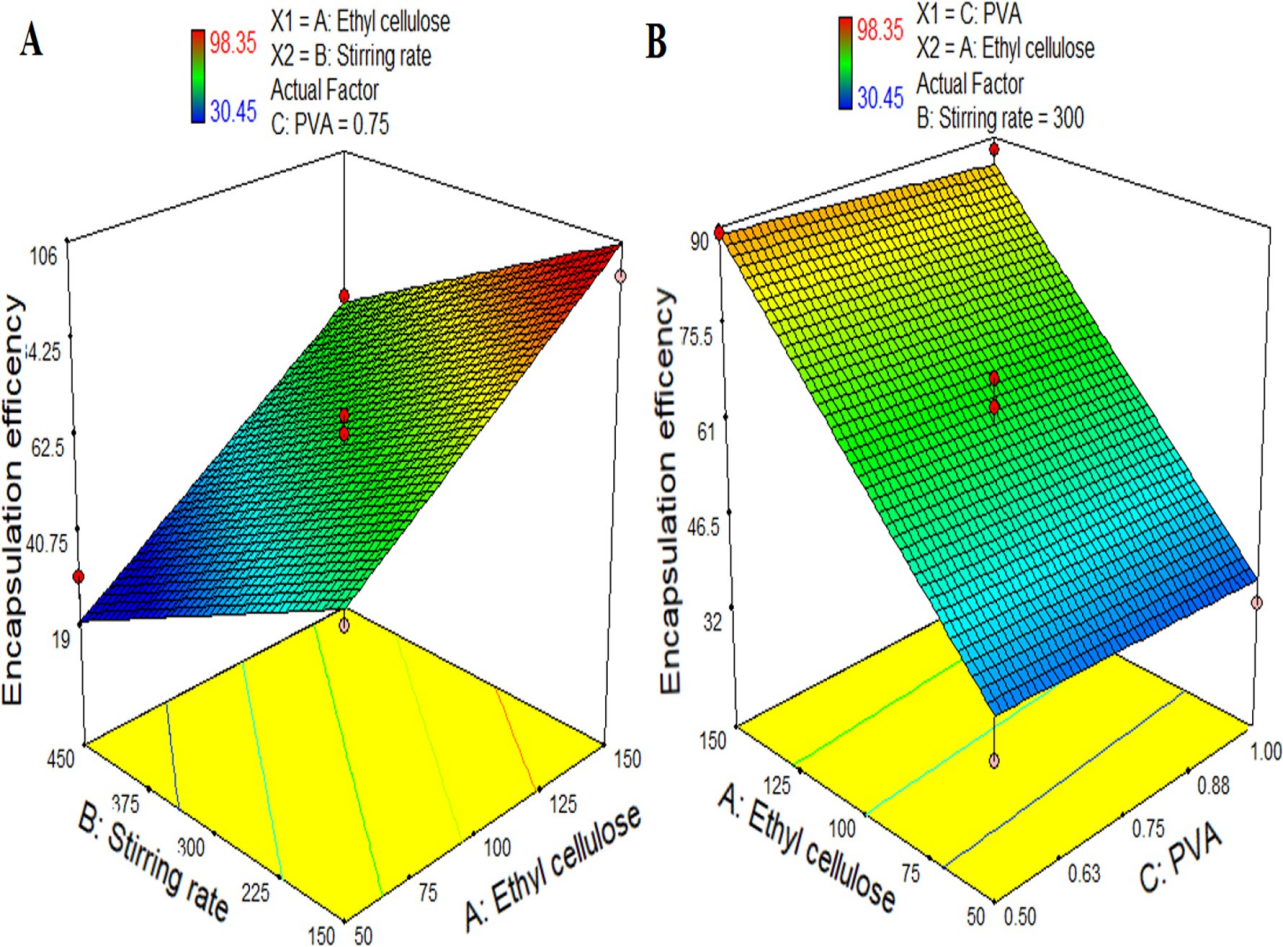
3-dimensional response surface graphs indicating (A) the effect of the concentration of polymer and stirring speed on EE, and (B) the effect of the surfactant (PVA) and polymer concentration on EE.

The direct method has been adopted to find out the percent EE of PTZ in the microspheres. Fig 2 indicates the effect of the polymer (ethyl cellulose) concentration, stirring speed, and surfactant (PVA) concentration on the EE of prepared microspheres with uniform drug contents. Formulation P12 showed maximum entrapment of PTZ (98.35%) while formulation P9 showed minimum encapsulation of PTZ (30.45%). It was evident from the ANOVA analysis and coefficients of the regression equation that the concentration of the polymer (X1) and stirring speed (X2) have a significant effect on the %EE of the microspheres. While the effect of surfactant concentration (X3) is insignificant on the %EE of the PTZ.

The quadratic expression relating the E.E (%) to the independent variables was as shown in Eq 10

$$\mathrm{Entrapment}\;\mathrm{Efficiency}\;(Y_{2})=62.73+24.60\;X_{1}-18.16\;X_{2}-1.55X_{3}-1.44\;X_{1}X_{2}-0.11\;X_{1}X_{3}-0.71\;X_{2}X_{3}-1.75\;X_{1}^{2}+1.99\;X_{2}^{2}-0.24\;X_{3}^{2}$$ (10)

The %EE of the formulations was found to decrease significantly with an increase in stirring speed (X2) because an increase in the stirring speed (X2) decrease the particle size of the microspheres which in turn cause a decrease in EE of the drugs within the system [42–44]. The %EE also depends upon the amount of ethyl cellulose (X1). By increasing the amount of ethyl cellulose (X1) the EE of the drugs increases. PTZ is highly lipophilic, therefore, it may also increase the %EE [45].

#### 3.1.3. Effect on dissolution rate/drug release

**Fig 3:**
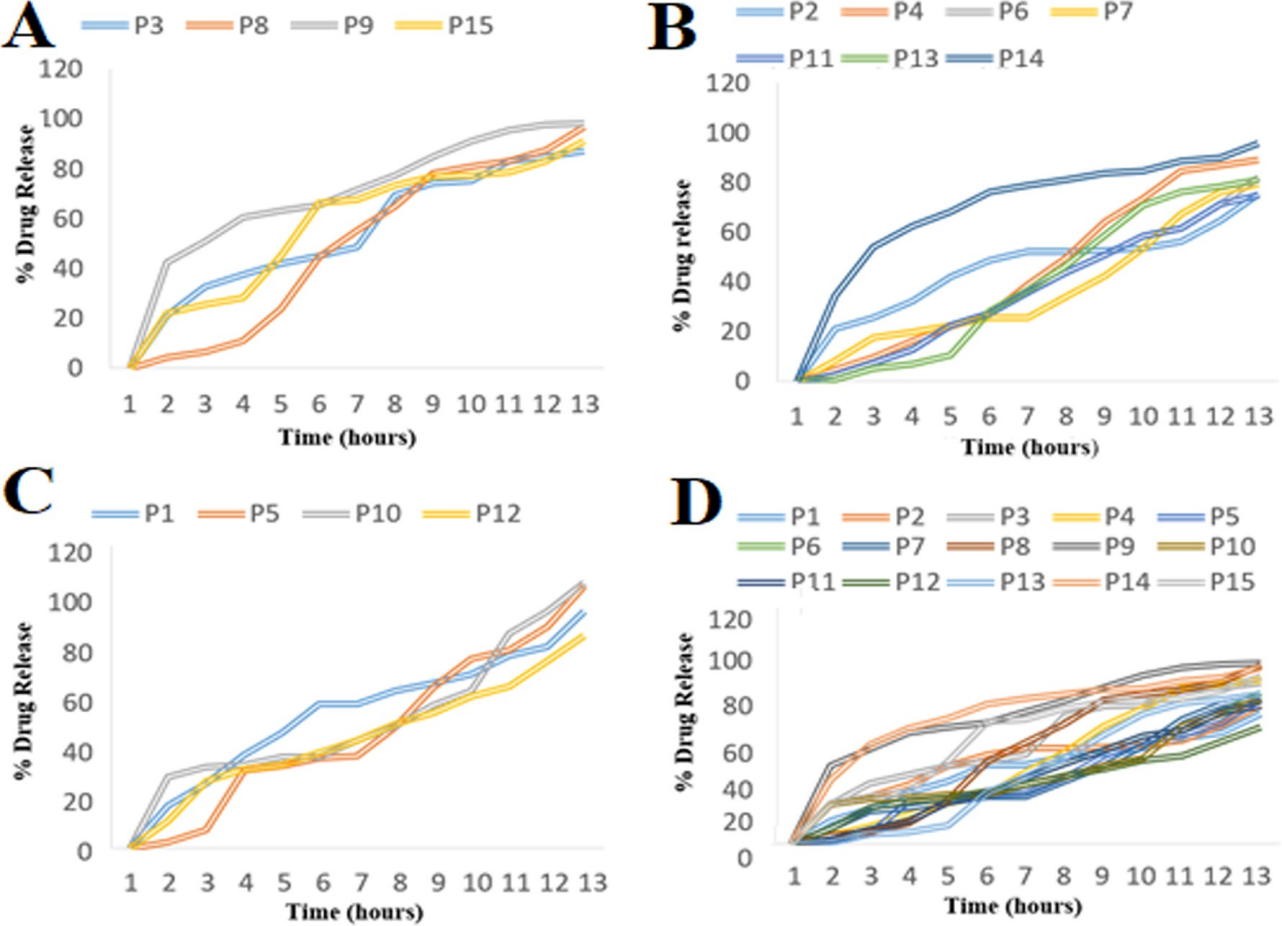
Drug release profiles of the microspheres prepared with (A) 50 mg of ethyl cellulose, (B) with 100 mg of ethyl cellulose, (C) with 150 mg of ethyl cellulose, and (D) of the all formulations.

**Fig 4:**
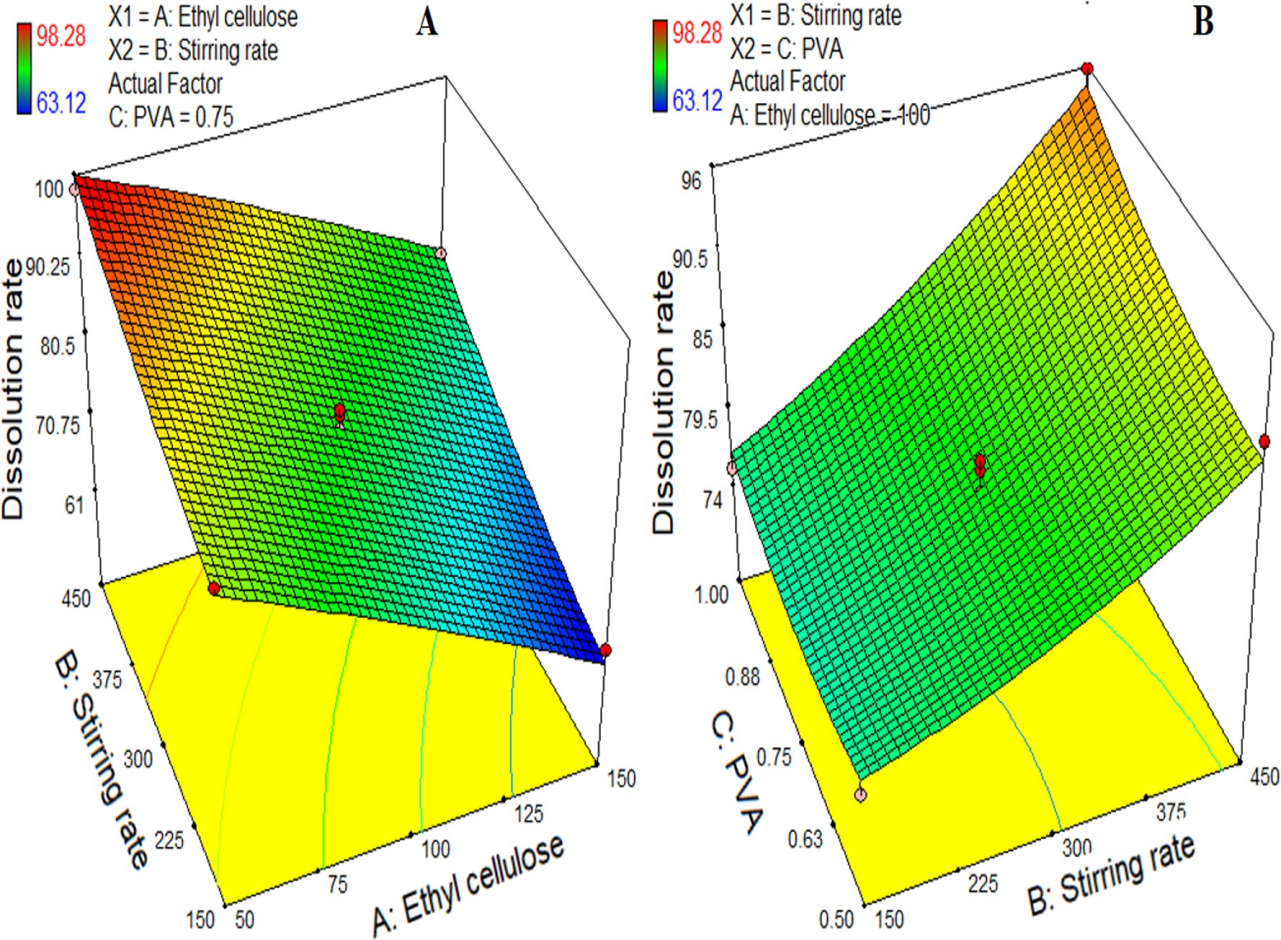
3-dimensional response surface graphs indicating (A) the effect of the concentration of polymer and stirring speed on dissolution rate, and (B) the effect of the surfactant (PVA) concentration and stirring speed on dissolution rate.

The *in-vitro* release studies were performed using Type II apparatus by paddle method. The perfect sink conditions were maintained through the experiments. Percent Cumulative amount of drug released was determined for individual formulations (Table 2) and indicated in Fig 3 by plotting Percent cumulative drug release versus time. The variation in the release of the drug corresponds to different independent variables was explained by the given quadratic expression (Eq 11) and presented by 3D contour graphs in Fig 4.

$$\mathrm{Dissolution}\;\mathrm{rate}\;(Y_{3})=80.89-11.60\;X_{1}+7.62\;X_{2}+1.82X_{3}+0.97\;X_{1}X_{2}-1.07\;X_{1}X_{3}+1.56\;X_{2}X_{3}-0.57\;X_{1}^{2}+1.35\;X_{2}^{2}+1.53\;X_{3}^{2}$$ (11)

The drug release from the PTZ from the microspheres showed a biphasic release behavior, indicating an initial burst release followed by the sustained release over a period of 12 h (Fig 3). The porous surface of the prepared microspheres may contribute to the initial release of PTZ by giving quick access of dissolution medium to the drug. While the later sustained release may be attributed by the presence of release retardant polymer ethyl cellulose in the form of the matrix that controls the penetration of the dissolution medium in the microspheres.

The release retardant polymer, ethyl cellulose (X1,) significantly affected the release of PTZ from microspheres. The formulations containing higher amounts of ethyl cellulose (Fig 3C), showed a more sustained release of PTZ that was due to the formation of a compact matrix of the microsphere and a relatively thicker layer of the polymer on the encapsulated drug [46]. In contrast, the decreased concentration of ethyl cellulose (X1) resulted in a quicker release of the drug [47]. The % w/v of PVA (X3) was found to have insignificant effects on the percent cumulative drug release of the drug from the microspheres. According to the reported literature, the hydrophilic polymer in combination with PVA provides quicker release but PVA alone does not cause faster drug release [48].

### 3.2. Optimization

The desirability approach has been employed for the selection of optimized formulation. Numeric optimization indicated the data of independent variables (X1, X2, and X3) used to prepare the optimized formulation with the desired outcomes (Y1, Y2, and Y3) indicated in Table 1. The optimized formulation was prepared in the triplicate manner through the optimal levels of the independent variables, suggested by the Design-Expert^®^. The predicted and actual values of dependent and independent variables were given in Table 3. The closeness in the predicted and actual values of these variables signifies the use of Design-Expert^®^ as a reliable optimization tool to formulate various micro and nano drug delivery carriers.

**Table 3:** Predicted and actual values of independent and dependent variables for the optimized formulation.

Polymer (mg)	Stirring speed (rpm)	Surfactant conc. (%)	Particle Size (nm)	%Entrapment Efficiency	Dissolution rate/Drug release	Desirability
	**Predcited formulation**					
88.82	150	1.0	97.52	73.86	79.37	0.96
	**Actual Optimized formulation**					
90	150	1.0	94.23±2.39	68.71±4.7	74.55±0.9	-------

All data is presented as mean±SD, (*n* = 3).

### 3.3. Morphological analysis of microsphere

**Fig 5:**
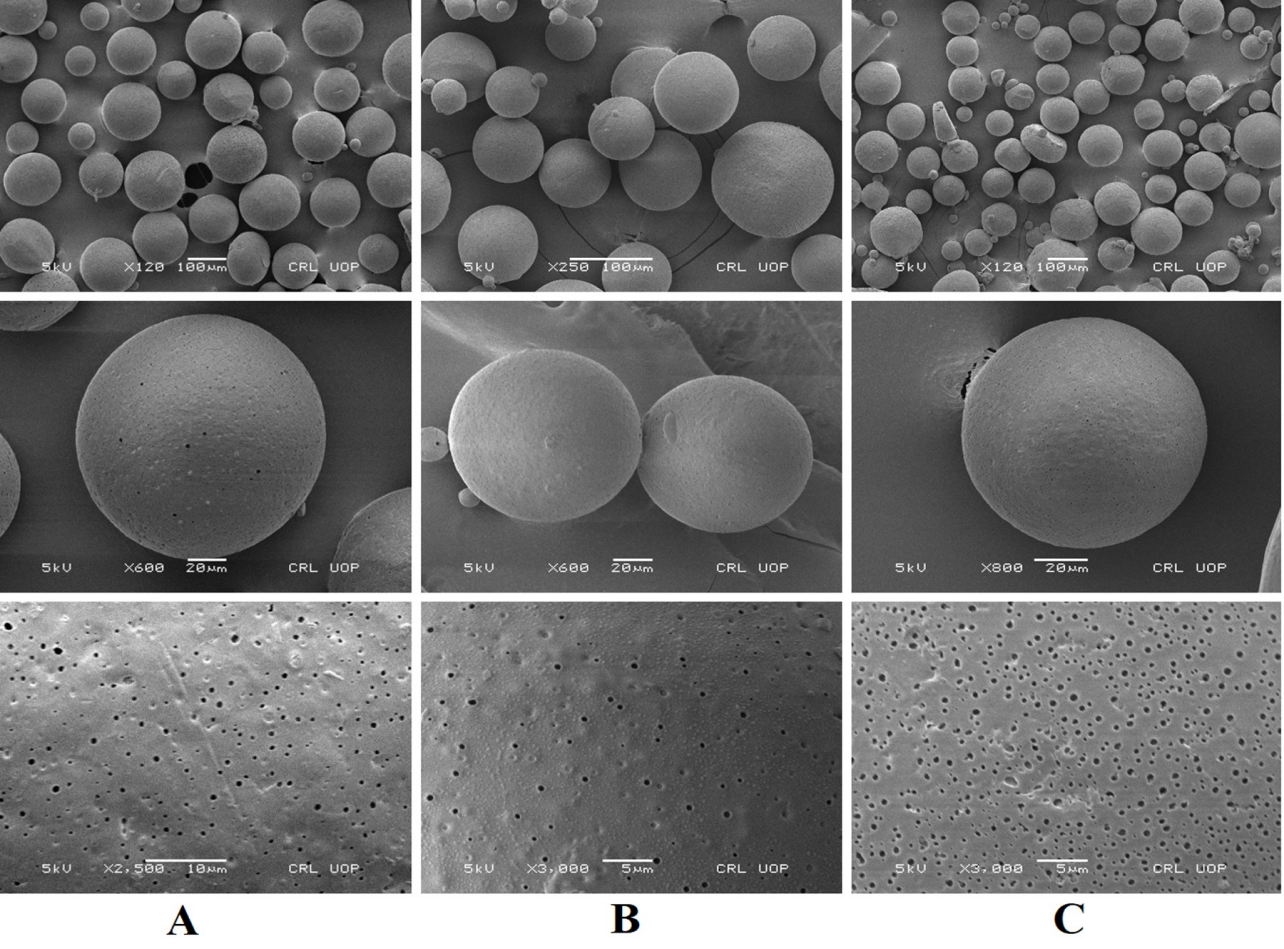
SEM analysis of Formulation P3 (A), Formulation P5 (B) and Formulation P10 (C) at different magnifications.

The field emission electron microscopy (JSM-5910, JEOL, Japan) was employed to evaluate the morphology of microspheres formulations (P3, P5, and P10). Fig 5 represents images with different magnifications and resolutions of the selected formulations (P3, P5 and P10). The microspheres were found entirely spherical with the porous surface following the previous literature [49].

### 3.5. Percent yield

The percent yield was more than 66% in all the microsphere formulations. The P7 formulation provided the highest yield of 98.67±1.00 while the P3 yielded the lowest of 66.00±2.08 (Table 2).

### 3.6. Drug release and kinetics

Various kinetic models, including zero order, first order, Higuchi and Korsmeyer-Peppas models, can best indicate the order and release mechanisms. The values of correlation coefficient (R^2^) were used to identify the most appropriate model to explain the microspheres’ drug release behavior. The correlation coefficient (R^2^) of all the formulations were given in Table 4. It was evident from the data that the PTZ release from the microspheres follows the Higuchi model with higher R^2^ values. The diffusional constant (*n*) in the Korsmeyer-Peppas equation was used to predict the drug release mechanism. Here, the value of n is different for different formulations that describe the release followed n = 0–0.5 as Fickian diffusion, n = 0.5–1.0 for non-Fickian transport.

**Table 4:** Values of correlation co-efficient of different kinetics models on release data.

Formulation Code	Zero Order	First Order	Higuchi Model	Krosmeyer- Peppas Model	
	R^2^			R^2^	*N*
P1	0.0105	0.9701	0.8653	0.9664	0.335
P2	-0.4706	0.9301	0.7053	0.9539	0.270
P3	-0.0496	0.9352	0.8535	0.9628	0.331
P4	10.616	0.9789	0.9324	0.9389	0.560
P5	0.7477	0.8532	0.9600	0.9646	0.553
P6	0.7553	0.9801	0.9644	0.9718	0.563
P7	0.7587	0.9502	0.9564	0.9641	0.566
P8	0.5853	0.831	0.9049	0.9050	0.503
P9	-1.1685	0.8318	0.4898	0.9865	0.214
P10	0.5212	0.8098	23.550	0.9513	0.447
P11	8.658	0.9630	23.550	0.9561	0.535
P12	0.3434	0.7077	0.9569	0.9891	0.396
P13	0.7360	0.9702	0.8939	0.9088	0.595
P14	-1.5950	0.8454	0.2762	0.9530	0.184
P15	0.7651	0.8766	0.7667	0.9098	0.311

### 3.7. FTIR analysis

**Fig 6:**
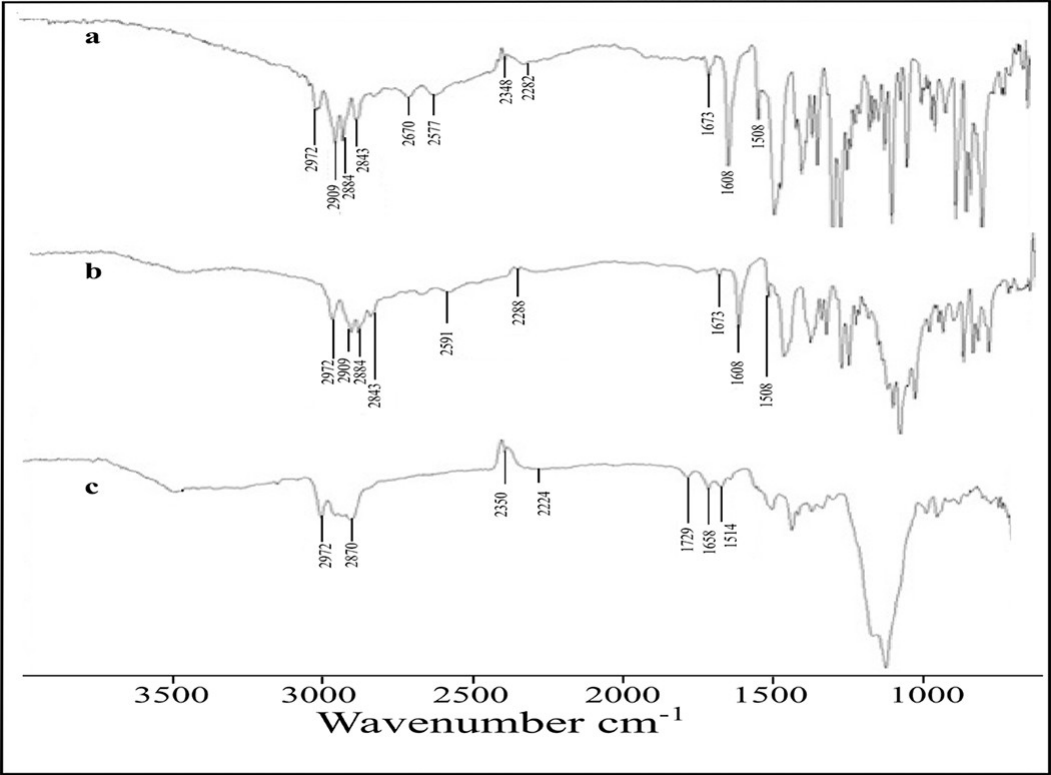
FTIR spectra of Pentazocine (a), Physical mixture (b), and optimized microsphere formulation (c).

ATR-FTIR analysis was employed to find any possible interaction among the different formulation components. The lack of any significant change also indicated the compatibility of all the structural components. The FTIR spectra of pure PTZ, physical mixture and prepared formulation have been depicted in Fig 6. [50]. The PTZ showed major FTIR peaks at 2884 cm^-1^, 2909 cm^-1^ and 2972 cm^-1^ (Fig 6A) associated with carboxyl (–COOH) group stretching, 2851 cm^-1^ represented alkane (-CH) group stretching, 2288 cm^-1^ showed stretching of nitrile (-CN) group, 1674 cm^-1^ depicted aliphatic alkene (C = C) group stretching, 1607 cm^-1^ revealed amide (-CONH) group bending and peak at 1508 cm^-1^ indicating aromatic (C = C) stretching [51, 52]. The physical mixture (Fig 6B) showed various intact peaks at 1508 cm^-1^, 1608 cm^-1^, 1674 cm^-1^, 2288 cm^-1^, 2591 cm^-1^, 2844 cm^-1^, 2884 cm^-1^, 2909 cm^-1^ and 2972 cm^-1^, respectively, suggesting the compatibility of various components and lack of any interaction among them. Whereas, the FTIR spectra of formulation (Fig 6C) showed significant peaks at 2972 cm^-1^ 2870 cm^-1^, 1729 cm^-1^, 1658 cm^-1^, and 1614 cm^-1^ that revealed the successful encapsulation of PTZ in the prepared microspheres [12].

### 3.8. Thermal analysis

**Fig 7:**
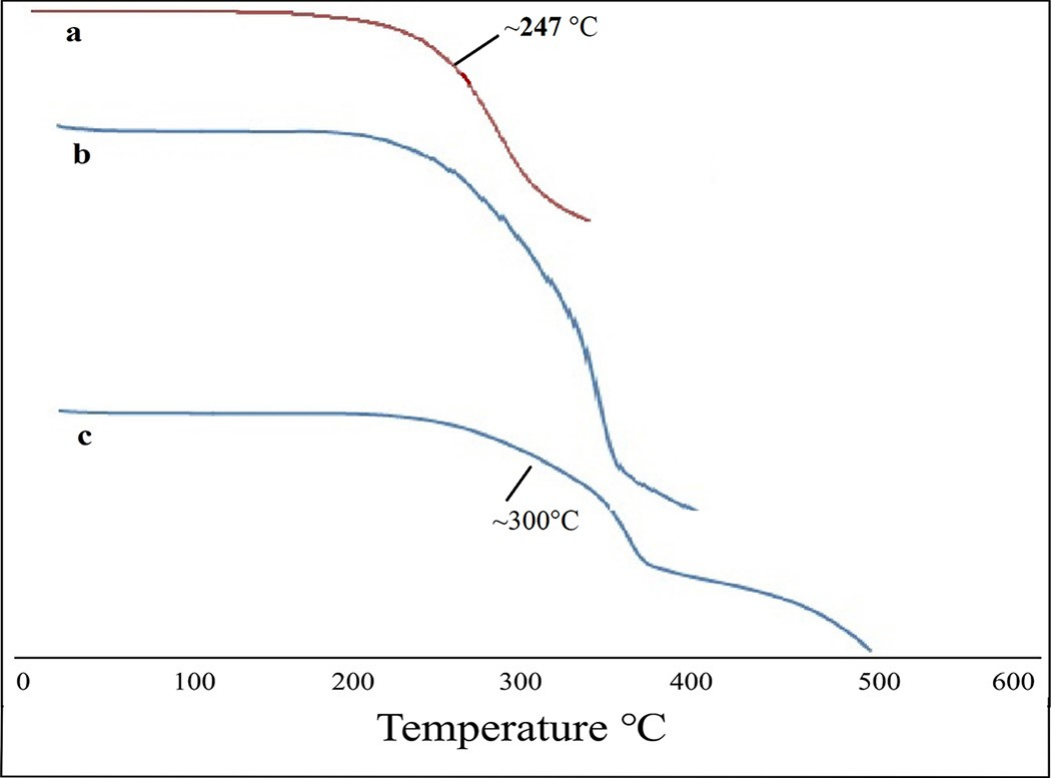
Thermogravimetric analysis of Pentazocine (a), Physical mixture (b), and optimized microsphere formulation (c).

TGA of PTZ, ethyl cellulose, physical mixture and formulation was performed by TG analyzer (SDT Q600, TA Instrument Co., Ltd., America) to determine their degradation temperature. TGA revealed a slight weight loss of the drug at the melting point of 247°C while the physical mixture showed signs of degradation around about 300°C (Fig 7B). Microspheres also exhibited decomposition after 300°C, which shows the physical stability of the system. The absence of the melting peak of PTZ at Σ247°C is due to the conversion of the crystalline form of the drug to an amorphous form during the solvent evaporation process which complies with the previous literature [53, 54].

### 3.9. Powder X-ray diffraction (PXRD) studies

**Fig 8:**
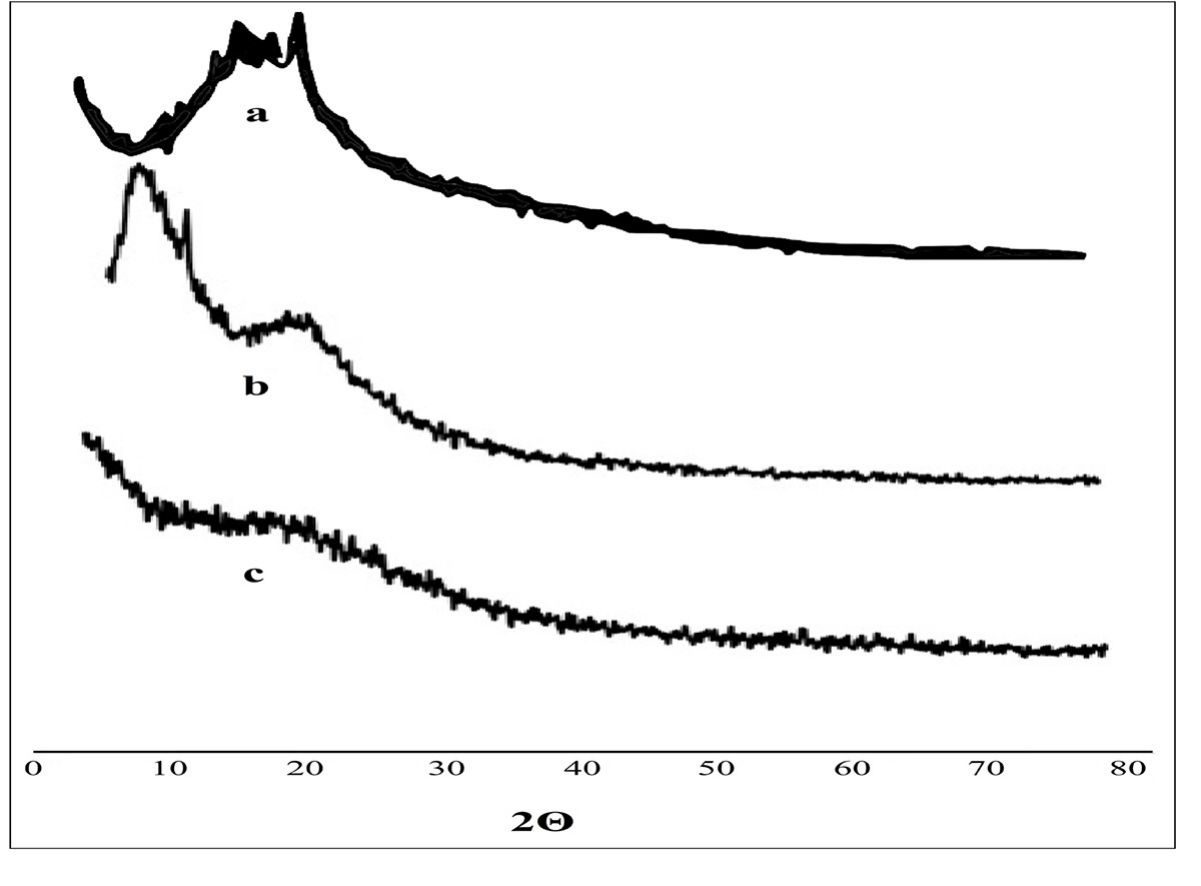
Powder x-ray diffractograms of Pentazocine (a), Physical mixture (b), and optimized microsphere formulation (c).

The PXRD spectrums of the pure PTZ, ethyl cellulose and microspheres formulations were recorded and shown in Fig 8. The diffractogram of pure PTZ indicated the characteristic sharp peak at 21° (Fig 8A). The broad diffused peaks were presented in the diffractogram of the physical mixture indicating the amorphous nature of the ethyl cellulose (Fig 8B) [27]. However, the diffuse peaks were observed in the diffractogram of the formulation (Fig 8C) which confirms the amorphous nature of the PTZ encapsulated in the microspheres [55, 56].

## 4. Conclusion

The PTZ loaded microspheres were successfully prepared and optimized by O/W emulsion solvent evaporation technique using ethyl cellulose as a carrier and PVA as an emulsifier. The important formulation parameters including particle size, entrapment efficiency and dissolution rate have been optimized and analyzed against different independent variables by three levels of three factorial box-Behnken designs. The developed microspheres were further characterized as spherical with a porous surface, high percentage yield and other physicochemical properties by TGA, PXRD and FTIR analysis. The optimized formulation showed sustained release of PTZ that improves the performance of the DDS. Overall, these polymeric microspheres provide a versatile platform for the delivery of different therapeutic moieties for various pathological conditions.

## Supporting information

S1 File (DOCX)

S1 Graphical abstract (TIF)
