# Supplementary material for: Statistically optimized pentazocine loaded microsphere for the sustained delivery application: Formulation and characterization
Source: PLoS One. 2021 Apr 30;16(4):e0250876. doi: 10.1371/journal.pone.0250876 (PMC8087016; doi:10.1371/journal.pone.0250876)
Supplement: S1 File — (DOCX) [file pone.0250876.s001.docx]

**Data file for optimization**

**ANOVA for Response Surface Quadratic Model Particle size**

**Response** **1** **Particle size**
 **Analysis of variance table [Partial sum of squares - Type III]**
 **Sum of** **Mean** **F** **p-value**
 **Source** **Squares** **df** **Square** **Value** **Prob > F**

Model 9497.56 9 1055.28 107.19 < 0.0001 significant

*A-Ethyl cellulose* *6232.86* *1* *6232.86* *633.13* *< 0.0001*
 *B-Stirring rate* *2768.05* *1* *2768.05* *281.18* *< 0.0001*
 *C-PVA* *235.34* *1* *235.34* *23.91* *0.0045*
 *AB* *60.53* *1* *60.53* *6.15* *0.0559*
 *AC* *4.75* *1* *4.75* *0.48* *0.5181*
 *BC* *42.84* *1* *42.84* *4.35* *0.0914*
 *A2* *78.77* *1* *78.77* *8.00* *0.0367*
 *B2* *27.19* *1* *27.19* *2.76* *0.1574*
 *C2* *42.34* *1* *42.34* *4.30* *0.0928*
 Residual 49.22 5 9.84
 *Lack of Fit* *42.49* *3* *14.16* *4.21* *0.1980* *not significant*
 *Pure Error* *6.73* *2* *3.37*
 Cor Total 9546.78 14

**ANOVA for Response Surface Quadratic Model for Dissolution rate**

**Response** **3** **Dissolution rate**

 **Analysis of variance table [Partial sum of squares - Type III]**
 **Sum of** **Mean** **F** **p-value**
 **Source** **Squares** **df** **Square** **Value** **Prob > F**

Model 1600.87 9 177.87 58.93 0.0002 significant

*A-Ethyl cellulose* *1075.55* *1* *1075.55* *356.34* *< 0.0001*
 *B-Stirring rate* *464.21* *1* *464.21* *153.80* *< 0.0001*
 *C-PVA* *26.50* *1* *26.50* *8.78* *0.0314*
 *AB* *3.74* *1* *3.74* *1.24* *0.3160*
 *AC* *4.56* *1* *4.56* *1.51* *0.2738*
 *BC* *9.70* *1* *9.70* *3.21* *0.1330*
 *A2* *1.21* *1* *1.21* *0.40* *0.5547*
 *B2* *6.76* *1* *6.76* *2.24* *0.1948*
 *C2* *8.68* *1* *8.68* *2.87* *0.1508*
 Residual 15.09 5 3.02
 *Lack of Fit* *12.28* *3* *4.09* *2.92* *0.2657* *not significant*
 *Pure Error* *2.81* *2* *1.40*
 Cor Total 1615.96 14

Formuation of Calibration curve

**Standard curve Analysis**

| **Conc. (µg/ml)** | **Take** | **Make** | **Conc. (µg/ml)** | **Abs** |
| --- | --- | --- | --- | --- |
| 10 | 0 | 10 | 0 | 0.000 |
| 10 | 1 | 10 | 1 | 0.012 |
| 10 | 2 | 10 | 2 | 0.022 |
| 10 | 4 | 10 | 4 | 0.045 |
| 10 | 5 | 10 | 5 | 0.049 |
| 10 | 6 | 10 | 6 | 0.053 |
| 10 | 7 | 10 | 7 | 0.066 |
| 10 | 8 | 10 | 8 | 0.074 |
| 10 | 9 | 10 | 9 | 0.081 |
| 10 | 10 | 10 | 10 | 0.091 |
| 20 | 7.5 | 10 | 15 | 0.123 |
| 20 | 10 | 10 | 20 | 0.170 |
