# Supplementary figures and images for: Statistically optimized pentazocine loaded microsphere for the sustained delivery application: Formulation and characterization
Source: PLoS One. 2021 Apr 30;16(4):e0250876. doi: 10.1371/journal.pone.0250876 (PMC8087016; doi:10.1371/journal.pone.0250876)

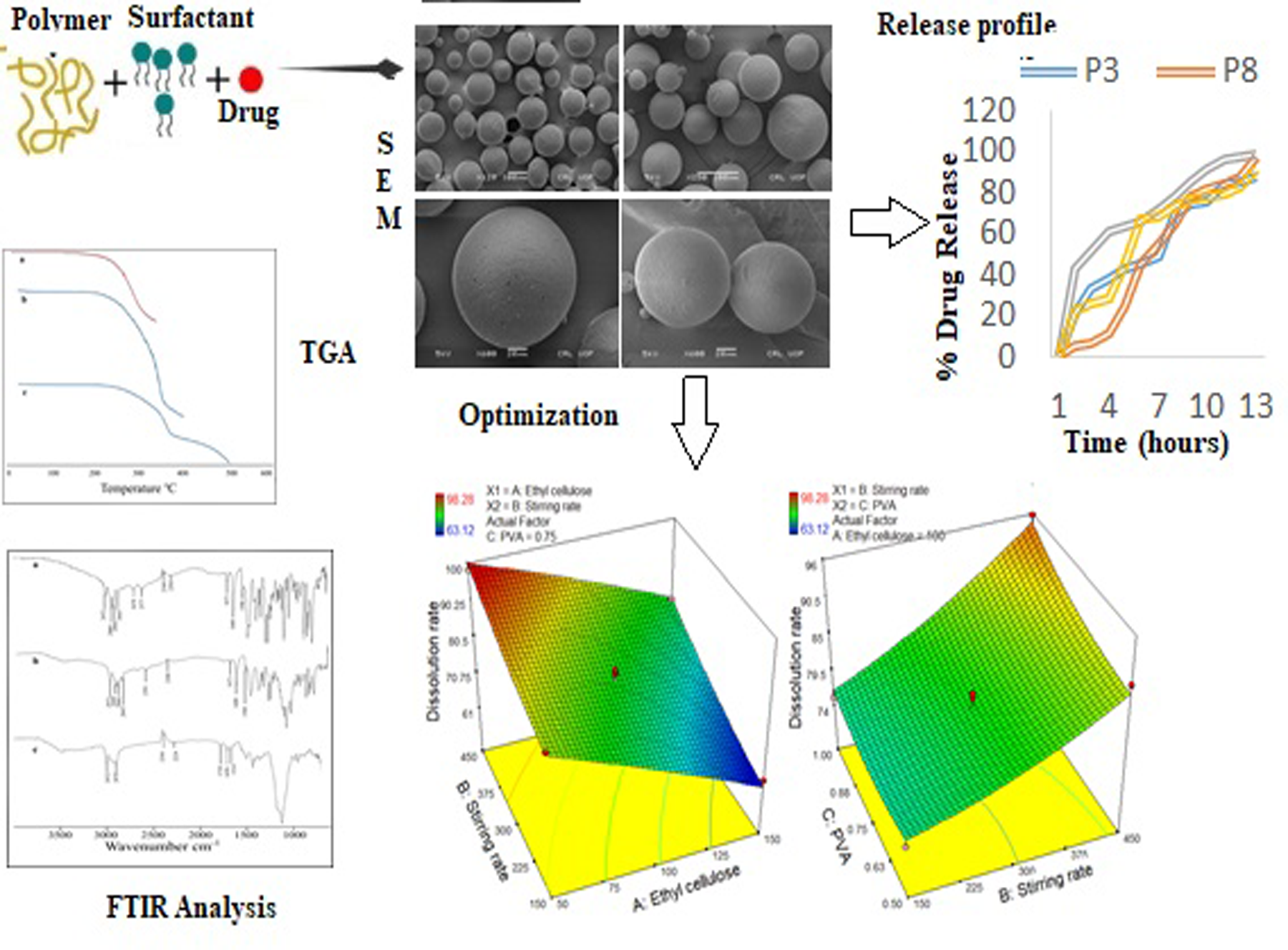

Supplement: S1 Graphical abstract — (TIF) [file pone.0250876.s002.tif]
